# Gastrointestinal Bleeding During Long-Term Left Ventricular Assist Device Support: External Validation of UTAH Bleeding Risk Score

**DOI:** 10.3390/jcdd12030105

**Published:** 2025-03-19

**Authors:** Giuseppe Vadalà, Cristina Madaudo, Alessandra Fontana, Vincenzo Sucato, Gioele Bicelli, Laura Maniscalco, Antonio Luca Maria Parlati, Giovanna Panarello, Sergio Sciacca, Michele Pilato, Manlio Cipriani, Alfredo Ruggero Galassi

**Affiliations:** 1 Division of Cardiology, University Hospital “P. Giaccone”, Via Del Vespro 129, 90100 Palermo, Italy; 2 Department of Health Promotion, Mother and Child Care, Internal Medicine and Medical Specialties, University of Palermo, 90127 Palermo, Italy; 3 Institute of Transplant and Highly Specialized Therapies (ISMETT) of Palermo, 90127 Palermo, Italy; 4 Department of Advanced Biomedical Sciences, University of Naples Federico II, 80131 Naples, Italy

**Keywords:** end-stage heart failure, left ventricular assist device, gastrointestinal bleeding

## Abstract

Background: Gastrointestinal bleeding (GIB) is a common complication of left ventricular assist device (LVAD) support. The UTAH bleeding risk score (UBRS) is the only dedicated GIB prediction model, but its efficacy has not been confirmed in an external validation cohort. Furthermore, the reliability of other bleeding risk scores, such as ARC-HBR and HASBLED, has never been tested in this specific population. This study aims to validate the UBRS and compare its accuracy with the ARC-HBR and HASBLED scores. Methods: Major adverse events (MAEs) and bleeding events of 75 consecutive patients who had undergone LVAD implantation between 2010 and 2021 at a referral hospital for a heart transplant were retrospectively analyzed. The accuracy of the UBRS, ARC-HBR and HASBLED scores was evaluated using a ROC curve model. Results: At a mean follow-up of 905.9 ± 724 days, 58 (77.3%) patients had an MAE and 28 (37.3%) had a major bleeding event. Out of the 39 major bleeding events, the majority were GI (43%) and intracranial bleeding (33.3%). Compared with patients without major bleeding, those who experienced major bleeding showed a lower survival probability, regardless of the nature of the bleeding (GIB vs. other bleeding events). The UBRS effectively stratified the bleeding risk with an AUC of 0.86. In contrast, the ARC-HBR and HASBLED scores demonstrated lower discriminatory power, with AUCs of 0.61 and 0.52, respectively. Conclusions: UBRS accuracy was confirmed in our study population. Gastrointestinal bleeding is a common life-threatening complication and one of the main causes of re-hospitalization during VAD support, leading to a lower patient survival probability.

## 1. Introduction

Although ventricular assist device (VAD) therapy has improved the survival of patients with advanced heart failure compared with medical therapies alone, VAD recipients frequently develop life-threatening complications, such as bleeding, thromboembolism, and pump thrombosis [1,2].

Major bleeding represents the most common cause of re-hospitalization, with an estimated incidence ranging between 40% and 60%. Half of them are gastrointestinal bleeding (GIB) [3,4,5,6,7]. Given the prognostic impact of major bleeding complications, a GIB risk assessment might have essential implications for candidate selection, the informed consent process, and individualizing post-implant therapeutic strategies. However, an accurate definition of the GIB risk is challenging in this setting. The only existing dedicated predicting model, UTAH bleeding risk score (UBRS), could not predict GIB in an external validation cohort of patients [8,9]. Furthermore, the two most popular bleeding risk scores, the ARC-HBR (Academic Research Consortium for High Bleeding Risk) and HASBLED, designed, respectively, for patients undergoing percutaneous coronary intervention and for those with atrial fibrillation, to the best of our knowledge, have not yet been tested in a consecutive series of VAD patients [10,11].

The aims of the study were the following: (1) to validate the UBRS in our cohort of continuous-flow VAD recipients and to compare its accuracy with that of the ARC-HBR and HASBLED, respectively, and (2) to investigate the clinical impact of major bleeding, both GIB and non-GIB.

## 2. Materials and Methods

Seventy-five patients who had undergone VAD implantation between November 2010 and December 2021 were enrolled at a tertiary referral hospital for transplant in this retrospective study cohort. The local Ethics Committee approved the study, and all patients were provided the informed consent document. VAD therapy was indicated as the bridge to transplant (BTT), destination therapy (DT), and as the bridge to candidacy (BTC) in patients deemed to be unsuitable for cardiac transplant. The local Heart Team established the patient-based treatment strategy. The two continuous-flow LVAD devices implanted were HeartWare (HVAD—Medtronic, Minneapolis, MN, USA) and HeartMate III (HM III—Abbott Laboratories, Lake Bluff, IL, USA). All the patients’ data, including clinical characteristics, laboratory and instrumental findings, medications, and adverse events, were derived from the electronic records and were compared with patients who had had a GIB and those who had not. All patients underwent an echocardiographic assessment the week before the LVAD was implanted; the left ventricular ejection fraction (LVEF) and right ventricle function were visually estimated. Before the VAD implantation, the patients underwent cardiac catheterization for the invasive assessment of right atrial pressure, pulmonary arterial pressure, pulmonary capillary wedge pressure, cardiac index, pulmonary vascular resistance, systemic blood pressure, and right ventricular (RV) stroke work index.

### 2.1. Risk Stratification

The following risk stratification models were utilized in this research to categorize patients based on their bleeding risk profile:

(1) The HAS-BLED score evaluates the 1-year risk of major bleeding in patients with atrial fibrillation and is calculated by assigning 1 point for each of the following factors: hypertension (systolic blood pressure > 160 mmHg), abnormal renal (creatinine ≥ 2.26 mg/dL, dialysis, or kidney transplant) or liver function (cirrhosis or significant hepatic impairment), stroke history, previous major bleeding or predisposition, labile INR (unstable or high INR if on warfarin), age ≥ 65 years, the use of drugs (antiplatelets and NSAIDs), or excessive alcohol consumption. The total score ranges from 0 to 9, classifying patients into low risk (0–2 points) and high risk (≥3 points), the latter indicating an increased likelihood of major bleeding events requiring closer monitoring [11].
(2) The ARC-HBR (Academic Research Consortium for High Bleeding Risk) classification identifies patients at high risk of bleeding, particularly in those undergoing percutaneous coronary intervention (PCI). Patients are considered at a high bleeding risk if they meet at least one major criterion or two minor criteria. Major criteria include previous major bleeding within six months or recurrent bleeding, severe anemia (Hb < 11 g/dL) or transfusion dependency, thrombocytopenia (platelet count < 100,000/μL), chronic kidney disease (CrCl < 30 mL/min), active malignancy with a bleeding risk, and stroke with hemorrhagic transformation or severe disability. Minor criteria include age ≥ 75 years, moderate anemia (Hb 11–12.9 g/dL in men, 11–11.9 g/dL in women), chronic kidney disease (CrCl 30–59 mL/min), long-term oral anticoagulation therapy, a history of recurrent or spontaneous gastrointestinal bleeding, and moderate thrombocytopenia (100,000–149,000/μL) [10].
(3) The UBRS (UTAH bleeding risk score) is calculated by attributing 1 or 2 points to specific variables: age > 54 years (1 point), coronary artery disease (1 point), chronic kidney disease (1 point), severe right ventricular (RV) dysfunction (1 point), mean pulmonary artery pressure < 18 mmHg (2 points), glucose > 107 mg/dL (1 point), and a history of previous bleeding (2 points). Based on the total score (ranging from 0 to 9), patients are stratified into low risk (0–1 point), intermediate risk (2–4 points), and high risk (5–9 points) [8].

### 2.2. Statistical Analysis

Continuous variables were expressed as means ± standard deviations (SDs), medians [25th, 75th percentiles] or *n* (%). Counts and percentages denote categorical variables. All the variables recorded were compared between the two study groups (GIB and control group without GIB). Student’s *t*-test or Mann–Whitney test, as appropriate, was used to compare the continuous variables of the two study groups; proportions were compared using the χ^2^ test and Fisher’s exact test. A Kaplan–Meier survival analysis was performed to measure the number of subjects free from events or who survived after the intervention over the study period. Univariable and multivariable Cox regression analyses were performed to identify factors independently associated with GIB in the study population.

Furthermore, the performance of the UBRS, ARC-HBR and HASBLED scores in predicting gastrointestinal bleeding was evaluated using a ROC curve by a Wilson–Brown hybrid method for the analysis. A two-sided *p*-value < 0.05 was considered statistically significant for single tests, whereas for multiple testing, the significance level was adjusted using the Bonferroni correction. All statistical analyses were performed using R studio software (version 1.4.1103 2009–2021 RStudio, PBC) and IBM SPSS (version 29.0.2.0 (20)).

### 2.3. Definitions

Bleeding events were defined as minor or major according to the Bleeding Academic Research Consortium (BARC) definition [12]. A GI bleed was defined as overt bleeding within the gastrointestinal tract (melena, hematochezia, hematemesis, and coffee-ground emesis). According to the timing of occurrence, GIB events were classified as early (within 30 days from VAD implantation) and late (after 30 days from VAD implantation). Device thrombosis was defined by the demonstration of a blood clot within any component of VAD; the thrombus could either originate from the left atrium or ventricle or from right-sided cardiac chambers through a septal defect [13].

### 2.4. Antithrombotic Therapy

After the device was implanted, all patients received bridging intravenous unfractionated heparin. As patients became able to tolerate oral medications, they switched from heparin to warfarin, with a recommended target international normalized ratio (INR) between 2 and 3. In case of thrombotic events at follow-up, acetylsalicylic acid (ASA) was added to anticoagulant therapy with a recommended dose of 100 mg daily. In case of major bleeding, the warfarin reversal was obtained by a four-factor prothrombin complex concentrate (4F-PCC).

## 3. Results

### 3.1. Clinical Characteristics

Patients’ characteristics are reported in Table 1. The mean age was 57.8 ± 8.9 years. All patients were in NYHA class ≥ 3. Ischemic cardiomyopathy was the most frequent heart failure etiology (48%), followed by dilatative cardiomyopathy (38.6%). VAD implantation was performed according to the following indications: BTT in 46.6% of patients, BTC in 34.8%, and DT in 18.6%.

All echocardiographic and right catheterization records are reported in Supplementary Table S1.

### 3.2. Procedural Characteristics

Table 2 shows the VAD procedural characteristics in detail. Most patients (93%) received the HVAD, while the remaining received the HM III. In 96% of procedures, pharmacological inotropic support by epinephrine alone or combined with other agents was used to optimize the patient’s hemodynamic status. Furthermore, supplementary mechanical circulatory support (MCS) by IABP or ECMO was used in 48% and 2.6% of procedures, respectively. The transfusion of blood products and renal replacement therapy were necessary in 56% and 35% of patients, respectively.

### 3.3. Bleeding Risk Stratification

**Figure 1:**
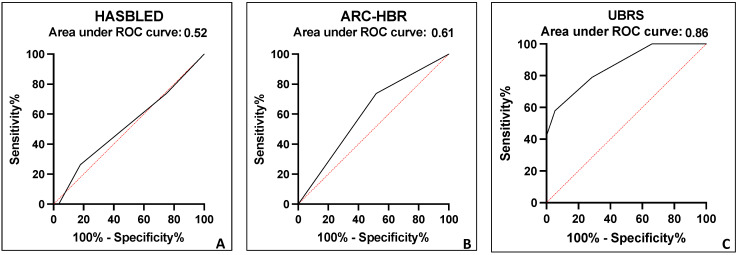
HASBLED (**A**), ARC-HBR (**B**), and UBRS (**C**) ROC curves. ARC-HBR = Academic Research Consortium for High Bleeding Risk; GIB = gastrointestinal bleeding; MB = major bleeding; HASBLED = Hypertension, Abnormal Renal/Liver Function, Stroke, Bleeding History or Predisposition, Labile INR, Elderly, Drugs/Alcohol Concomitantly; UBRS—UTAH bleeding risk score.

a. HASBLED score: A total of 73 (97.3%) of 75 patients had a HASBLED score ≤ 2. More precisely, 60 patients had a HASBLED value between zero and one, and 13 patients had a HASBLED score equal to two. The remaining two patients had a HASBLED score equal to three. None of the two patients with a score ≥ 3 developed a GIB, whereas 19 patients out of 73 with a score ≤ 2 (25%) had a GIB (*p* = 0.4). Therefore, the HASBLED score showed an area under the receiver operating characteristic curve of 0.52 (95% CI, 0.37–0.68) (Figure 1A).

b. ARC-HBR score: Eight patients (10.6%) were ranked as low risk, and sixty-seven patients (89.4%) had a high-risk ARC-HBR profile. GIB occurred in 18 of 67 (27%) high-risk patients and in one of the 8 (12.5%) low-risk patients (*p* = 0.3). Furthermore, out of the 56 patients who did not have GIB, 49 (87.5%) were ranked as high risk. Therefore, the ARC-HBR showed an area under the receiver operating characteristic curve of 0.61 (95% CI, 0.47–0.75) (Figure 1B).
c. UBRS: A total of 19 patients (25.3%) were ranked as low risk, 48 patients (64%) as intermediate risk, and 8 patients (10.7%) as high-risk, respectively. All eight patients classified as high risk had a GIB, whereas none of the nineteen patients classified as low risk had a GIB (*p* < 0.001). Out of the 48 patients classified as intermediate risk, 11 patients (23%) had a GIB, and 37 patients (77%) did not (*p* < 0.001). Finally, the performance of UBRS showed an area under the receiver operating characteristic curve of 0.86 (95% CI, 0.76–0.95) (Figure 1C).

### 3.4. Clinical Follow-Up

All the details are described in Table 3. At a mean follow-up of 1598 ± 1107 days, 29.3% of patients underwent heart transplants, and 54.6% died. Infections and bleeding were the most frequent causes of death. Indeed, twelve deaths were due to septic shock, two to SARS-COVID infections, and one to infective pneumonitis. The remaining ten deaths were due to fatal bleeding: eight due to intracranial hemorrhage and two to hemorrhagic shocks secondary to GIB, respectively. A total of 77% of patients had an MAE, 36% had multiple MAEs, and 37.3% of patients had major bleeding (Central illustration). Finally, the GIB and no GIB groups showed similar MAE rates (76.2% vs. 77.7%; *p* = 0.989).

### 3.5. Bleeding Complications and Outcome

**Figure 2:**
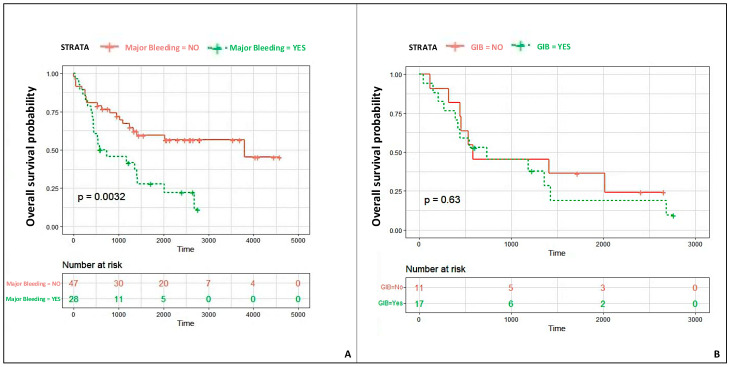
Time-to-event Kaplan–Meier curves: patients’ survival probabilities according to the major bleeding status (**A**) and according to the bleeding type (GIB vs. other major bleeding) (**B**).

Twenty-eight patients had major bleeding (37.3%); out of these, 43.5% were GI bleeding, and 33.3% were intracranial bleeding (Table 3 and Central illustration). Of note, 16% of patients had multiple major bleeding events. Furthermore, GIB patients were more likely to have multiple major bleeding events (GIB group vs. no-GIB group: 43% vs. 5.5%; *p* < 0.001). The GIB site was identified in 81% of the patients, with the small intestine in seven cases, followed by the stomach in six, the large intestine in three and the rectum in one. In the remaining four patients, the site of GIB was supposed to be a gastroscopy or colonoscopy but was non-conclusive. GIB etiologies were the following: eight (47.1%) due to arteriovenous malformation (AVM), four (23.5%) due to gastric ulcer, three (17.6%) due to colon diverticulosis, and two (11.8%) due to erosive gastritis. The duration of LVAD support was longer among patients with GIB than those without (968 ± 825 days vs. 881 ± 688 days; *p* < 0.001). At the time of the major bleeding event, the mean INR value was significantly higher in those with GIB than in those without (2.5 ± 0.9 vs. 1.5 ± 0.8; *p* < 0.001). The time-to-event Kaplan–Meier curves showed a significantly lower patient survival probability in patients with major bleeding than in the group without (*p* = 0.032). When stratified for the major bleeding type, patients who had a GIB showed a similar survival probability than those who had non-GI major bleeding event (Figure 2).

Furthermore, the patients who had major bleeding showed a higher INTERMACS class (*p* = 0.037), died more frequently (*p* = 0.012), and were less likely to undergo a heart transplant (*p* < 0.001) compared with those who did not have major bleeding. The multivariable analysis showed a reduced risk of major bleeding among patients who underwent a transplant (OR = 0.18, 95% CI = 0.03–0.68; *p* = 0.009).

## 4. Discussion

The main findings of the present study can be summarized as follows:

1. The UBRS was effective at predicting the GIB risk of LVAD recipients, while the ARC-HBR and HASBLED scores were not.
2. Patients on long-term VAD support showed a dual, dramatically high risk of developing MAEs and major bleeding complications.
3. Most major bleeding events were gastrointestinal and were associated with a significantly lower patient survival probability.

### 4.1. UBRS Validation and Applicability of Other Scores

The UBRS is the only dedicated score for defining the GIB risk of VAD patients. However, its validity is controversial since, in a recent study, UBRS did not receive external validation [9]. The authors explained such a result as a possible consequence of some existing differences between the population characteristics of the UBRS study and the validation cohort, for example, the proportions of non-ischemic vs. ischemic cardiomyopathy (55% vs. 35%) and of centrifugal vs. axial pump VAD (60% vs. 32%). Indeed, Boyle et al. showed that an ischemic etiology is a decisive risk factor for GIB complications in a multivariate analysis of 900 patients who underwent VAD implantation with the HeartMate II device (95% CI; hazard ratio: 1.35; *p* = 0.008) [14]. Conversely, the role of the centrifugal pump is less clear. In contrast, some evidence supports the fact that patients who had received pulsatile-flow LVAD have a reduced GIB incidence as compared with patients who received a continuous-flow LVAD; this finding was not confirmed in another retrospective registry where patients who were implanted with axial or centrifugal flow devices showed similar rates of bleeding complications [15,16]. Moreover, other factors may play a role in GIB occurrence. For example, it was postulated that non-pulsatile flow is a potent inducer of vascular remodeling that contributes to angiodysplasia and vascular frailty in patients with CF-LVAD [17]. Furthermore, in a study population of 595 patients implanted with HM II (HeartMate II), HM III, and HVAD (HeartWare LVAD) devices, the protective effects of pulsatility were recently highlighted in those patients with LVF > 30% who still maintain aortic valve opening and Lavare Cycle. The authors also hypothesized that flow pulsatility and aortic valve opening might avoid vascular remodeling, thereby decreasing the rate of gastrointestinal bleedings [18].

However, we have confirmed the external validity of the UBRS, although the proportion of patients who received a centrifugal pump VAD was lower in our registry than that of the UTHA population (32% vs. 93%). In contrast, the rates of ischemic vs. non-ischemic cardiomyopathy were similar (48% vs. 45%). Finally, we found that the ARC-HBR and HASBLED scores were not effective for a GIB risk assessment in our study population, probably because they have been trimmed based on other clinical settings, the complexity of which seems to be definitively lower compared to that of VAD recipients [11,12]. To the best of our knowledge, this was the first attempt to test the performance of these two scores in VAD patients.

### 4.2. Bleeding Events and Outcomes

Although the survival of patients with advanced heart failure has tremendously improved since the introduction of heart transplants and thanks to the implementation of VAD technology and more effective medical therapies, nowadays, VAD recipients still have a very high rate of thrombotic, bleeding, and infective complications [2,19].

Over the long term of 1598 ± 1107 days, the mortality rate of our study population was 54.6%; this result is consistent with one of the largest worldwide registries on long-term VAD support [20].

Furthermore, beyond infections that were the leading cause of morbidity, overall MAEs and major bleeding rates were as high as 77.3% and 37.3%, respectively. These data are in line with other previous studies [2,13,20].

Most of the major bleeding events were gastrointestinal, and all except one had a late onset. Whilst these results are consistent with a previous study by Goldstein et al., in which the GIB/patient/year rate reported was as high as 27%, the impact of GIB on patients’ outcomes is definitively less clear [13].

Indeed, Stulak et al. showed a correlation between GIB and subsequent adverse thromboembolic events defined as stroke, transient ischemic attack, hemolysis, or suspected or confirmed pump thrombosis [21]. The authors hypothesized that the reduction in the intensity of antithrombotic therapy following gastrointestinal bleeding could have increased the risk of thrombosis after the index GIB.

Conversely, Goldstein et al. found similar survival rates of VAD patients with GIB and those without (85% vs. 90.4%, respectively; *p* = 0.25) [13].

In our study, patients with major bleeding showed a significantly lower survival probability than those who had not experienced bleeding, regardless of whether the bleeding was GIB or not. Furthermore, the GIB group showed a similar rate of thrombotic complications to the no-GIB group. Regarding the latter point, we may hypothesize that the low number of thrombotic events secondary to the GIB resulted from combined prompt treatment at the bleeding site and the systematic use of four-factor prothrombin complex concentrate (4F-PCC) for warfarin reversal. Therefore, both actions reduced the time of non-adequate antithrombotic therapy superimposed by the hemorrhage. Indeed, the use of 4F-PCC has resulted in quicker and more predictable warfarin reversal in LVAD patients with no apparent higher risk of thromboembolism [22].

However, the great unpredictability of the outcome of patients who had major bleeding may depend on many other factors, and, therefore, it is still a great challenge [23]. For example, beyond warfarin reversal, which is possible in all types of major bleeding, a mini-invasive hemostatic intervention at the bleeding site is applicable and effective for most GIBs (e.g., gastro-duodenal ulcer or gastroesophageal varices). Still, it is not effective for many intracranial or thoracic bleeding events. Secondly, the very high burden of MAEs reported in both groups might have mitigated and confounded the real impact of GIB on patient outcomes.

However, the definition of the hemorrhagic risk is still a dominant concept of modern cardiology, encompassing different relevant areas such as arrhythmias, heart valve diseases, heart failure, and revascularization of patients with ischemic cardiomyopathy [24,25]. Therefore, any step forward in a bleeding risk assessment might help improve patients’ care. For example, we might speculate that some pre-existing conditions, like arteriovenous malformation (AVM) and colon diverticulosis, could be diagnosed before GIB occurrence by a preventive gastroscopy, colonoscopy, or other imaging tests, early at the time of the waiting list or later, during the follow-up after VAD implantation. In the hands of healthcare providers, this information might facilitate individualized follow-up strategies after a VAD implant to reduce the number of re-hospitalizations for GIB.

## 5. Limitations

We recognize many limitations of the present study. First, this study was based on a retrospective analysis with inherent limitations. Second, most patients were male (90%) and received an HVAD (93%); therefore, the results of our study might not apply to other cohorts of VAD patients. Third, although the management of GIB events generally followed the standard clinical practice, such as transfusions, cessation of antithrombotic therapy, antacid therapy, and diagnostic workup, the treatment was entirely left to the discretion of the caring physicians. Moreover, although higher INR values were observed in persons with GIB, the registry data did not allow us to determine if anticoagulant therapy was within an optimal range. Finally, the issue of the timing and intensity of the reintroduction of antithrombotic therapy following the resolution of a GIB event, still left at the discretion of the caring physician, might represent a potential bias.

## 6. Conclusions

The UBRS received external validation, despite some clinical and procedural differences between UTHA and our study population, such as the proportion of female patients, ischemic cardiomyopathy as an etiology of heart failure, and the proportion of continuous-flow VADs implanted. Gastrointestinal bleeding is a widespread life-threatening complication and one of the main causes of re-hospitalization during VAD support, leading to a lower patient survival probability. Individualizing the bleeding risk before VAD implantation could increase the accuracy of candidate selection and drive individualized post-implant strategies to prevent major bleeding.

## Figures and Tables

**Table 1:** Demographic and clinical characteristics.

	Overall (*n* = 75)	No GIB (*n* = 54)	GIB (*n* = 21)	*p*-Value
Age	57.8 ± 8.87	56.8 ± 9.7	60.2 ± 5.5	0.344
Female	7 (9.3)	7 (12.9)	0	N.S.
Hypertension	45 (60.0)	34 (63.0)	11 (52.0)	0.4
Hypercolesterolemia	36 (48.0)	25 (46.0)	11 (52.0)	0.6
Diabetes Mellitus	32 (43.0)	20 (37.0)	12 (57.0)	0.11
Chronic Kidney Disease (CKD)	42 (56.0)	28 (52.0)	14 (67.0)	0.2
Coronary Artery disease (CAD)	36 (48.0)	25 (46.0)	11 (52.0)	0.6
NYHA class				
III	44 (59.0)	34 (63.0)	10 (48.0)	0.2
IV	31 (41.0)	20 (37.0)	11 (52.0)	0.2
INTERMACS patient profile				
1	8 (10.6)	6 (11.1)	2 (9.5)	0.815
2	32 (42.6)	22 (40.7)	10 (47.6)	
3	20 (26.6)	16 (29.6)	4 (19.0)	
4	15 (20.0)	10 (18.5)	5 (23.8)	
Etiology of heart failure				
Ischemic	36 (48.0)	25 (46.3)	11 (52.4)	0.828
Dilatative Cardiomyopathy	29 (38.6)	21 (38.9)	8 (38.1)	1
Hypertrophic Cardiomyopathy	5 (6.6)	4 (7.4)	1 (4.8)	1
Chemotherapy Induced Cardiomyopathy	3 (4.0)	2 (3.7)	1 (4.8)	1
Myocarditis	1 (1.3)	1 (1.9)	0	N.S.
Valvular Cardiopathy	1 (1.3)	1 (1.9)	0	N.S.
Indication				
Bridge to Transplant (BTT)	35 (46.6)	26 (48.1)	9 (42.8)	0.942
Bridge to Candidacy (BTC)	26 (34.8)	18 (33.3)	8 (38.1)	
Destination Therapy (DT)	14 (18.6)	10 (18.5)	4 (19.1)	

Values are reported as means ± DSs or numbers (N) and percentages (%). BTT—bridge to Transplant; BTC—bridge to Candidacy; CAD—coronary artery disease; CKD—chronic kidney disease; DT—destination therapy; HVAD—HeartWare Ventricular Assist Device; NYHA—New York Heart Association.

**Table 2:** Procedural characteristics.

	Overall (*n* = 75)	No GIB (*n* = 54)	GIB (*n* = 21)	*p*-Value
Device Type				
HeartWare (HVAD)	70 (93.0)	51 (94.4)	19 (90.5)	0.615
HeartMate III (HM III)	5 (7.0)	3 (5.6)	2 (9.5)	
Inotropes/vasopressor				
Epinephrine	72 (96.0)	53 (95.0)	19 (100.0)	0.991
Norepinephrine	60 (80.0)	46 (82.0)	14 (74.0)	0.429
Dobutamine	16 (21.3)	13 (23.0)	3 (16.0)	0.497
Milrinone	32 (43.0)	23 (41.0)	9 (47.0)	0.631
Enoximone	2 (2.6)	2 (3.6)	0	N.S.
Vasopressin	6 (8.0)	3 (5.0)	3 (16.0)	0.166
Mechanical Circulatory Support				
IAPB	36 (48.0)	25 (44.6)	11 (57.9)	0.259
ECMO	2 (2.6)	2 (3.6)	0	N.S.
Transfusion of blood products	42 (56.0)	28 (52.0)	14 (67.0)	0.2
RBCs (n° of units)	3.1 ± 3.5	2.3 ± 3.4	2,4 ± 2.7	0.866
PLTs (n° of units)	2 ± 4.5	1.4 ± 3	3.6 ± 7	0.117
FFP (n° of units)	0.8 ± 1.6	0.8 ± 1.6	0.7 ± 1.3	0.838
Blood samples				
Hemoglobin (g/dL)	11.2 ± 2	11.4 ± 2	10.9 ± 2	0.350
Platelets (×10^3^/uL)	229 ± 87	227 ± 84	233 ± 95	0.802
eGFR, CKD-EPI (ml/min/1.73 m^2^)	67.8 ± 23	66 ± 21	72.6 ± 28.5	0.301
NT-proBNP (pg/mL)	6835 ± 9430	7527 ± 10,475	4798 ± 4700	0.317
Renal Replacement Therapy	26 (35.0)	18 (32.1)	8 (38.0)	0.697

Values are reported as means ± DSs, numbers (N), and percentages (%). ECMO—extracorporeal membrane oxygenation; eGFR—estimated glomerular filtration rate; FFP—fresh frozen plasma; IABP—intra-aortic balloon pump; NT-proBNP—N-terminal prohormone of brain natriuretic peptide; PLTs—platelets; RBCs—red blood cells; VAD—ventricular assist device.

**Table 3:** Adverse events at follow-up.

	Overall (*n* = 75)	No GIB (*n* = 54)	GIB (*n* = 21)	*p*-Value
Follow-up duration (days)	1598 ± 1107	1955 ± 1119	951 ± 1468	0.062
Duration of VAD support (days)	905.9 ± 724	881 ± 688	968 ± 825	<0.001
Transplant	22 (29.3)	18 (33.3)	4 (19.0)	0.269
Total number of MAEs	100	68	29	0.480
Patients with MAE	58 (77.3)	42 (77.7)	16 (76.2)	0.989
Patients with multiple MAEs	27 (36)	17 (31.5)	10 (47.6)	0.191
MAEs/patients	1.72	1.62	1.81	0.480
Death	41 (54.6)	28 (51.8)	13 (61.9)	0.598
Transient ischemic attack	3 (4)	3 (5)	-	N.S.
Ischemic stroke	16 (21.3)	11 (20.3)	5 (23.8)	0.759
Hospitalization for right ventricular failure	16 (21.3)	10 (18.5)	6 (28.5)	0.340
Hospitalization for VAD failure	16 (21.3)	12 (22)	4 (19)	0.763
VAD thrombosis	8 (10.6)	6 (11.1)	2 (9.5)	1
Total number of major bleeding events	39	13	26	
Patients with major bleeding	28 (37.3)	11 (20.3)	17 (81)	<0.001
Patients with multiple major bleeding events	12 (16)	3 (5.5)	9 (43)	<0.001
Major bleeding events/patient	1.43	1.18	1.53	<0.001
GIB	17 (43.5)	0	17 (65)	<0.001
Intracranial bleeding	13 (33.3)	7 (53.8)	6 (23)	0.054
Hemothorax	3 (7.7)	2 (15.3)	1 (3.8)	0.202
Nasopharyngeal hemorrhage	5 (12.8)	3 (23)	2 (7.7)	0.175
Colonstomy bleeding	1 (2.6)	1 (7.7)	-	0.151
Other complications				
VAD driveline infection	13 (17.3)	8 (14.8)	5 (23.8)	0.497
Sepsis	18 (24)	14 (25.9)	4 (19)	0.558
Wound dehiscence	17 (22.6)	15 (27.7)	2 (9.5)	0.124
Pneumonia	6 (8)	4 (7.4)	2 (9.5)	1
Pleural effusion	5 (6.7)	4 (7.4)	1 (4.7)	1
Acute renal failure	5 (6.7)	4 (7.4)	1 (4.7)	1
Acute cholecystitis	2 (2.6)	2 (3.7)	-	N.S.
Pancreatitis	1 (1.3)	1 (1.8)	-	N.S.
Pseudomembranous colitis	1 (1.3)	-	1 (4.7)	N.S.
Cognitive impairment	2 (2.6)	1 (1.8)	1 (4.7)	0.501
Pneumothorax	2 (2.6)	2 (3.7)	-	N.S.
Pneumoperitoneum	1 (1.3)	1 (1.8)	-	N.S.
Endocarditis	1 (1.3)	1 (1.8)	-	N.S.
SARS-CoV 2 infection	3 (4)	3 (5.6)	-	N.S.
Ventricular arrhythmia	20 (26.6)	14 (25.9)	6 (28.5)	0.563
Atrial arrhythmia	1 (1.3)	1 (1.8)	-	N.S.
Vascular access bleeding	1 (14.2)	1 (33.3)	-	N.S.
Nasopharyngeal hemorrhages	1 (14.2)	1 (33.3)	-	N.S.
Hemothorax	1 (14.2)	1 (33.3)	-	N.S.

Values are reported as means ± DSs, numbers (N) and percentages (%). GIB—gastrointestinal bleeding; MAEs—major adverse events; SARS-CoV2—severe acute respiratory syndrome coronavirus 2; VAD—ventricular assist device.

## Data Availability

The datasets used and/or analyzed during the current study are available from the corresponding author upon reasonable request. The custom code used for this study is available upon request from the corresponding author.

## Supplementary Materials

The following supporting information can be downloaded at www.mdpi.com/article/10.3390/jcdd12030105/s1: Table S1. Pre-LVAD implant instrumental findings.
