# Supplementary material for: Gastrointestinal Bleeding During Long-Term Left Ventricular Assist Device Support: External Validation of UTAH Bleeding Risk Score
Source: J Cardiovasc Dev Dis. 2025 Mar 19;12(3):105. doi: 10.3390/jcdd12030105 (PMC11943341; doi:10.3390/jcdd12030105)
Supplement: Supplementary file 1 [file jcdd-12-00105-s001.zip › jcdd-3437495-supplementary.pdf]

Supplementary table 1. Pre-LVAD implant instrumental findings.

	Overall (N = 75)	No GIB (N = 54)	GIB (N = 21)	p-value
**Echocardiography (before implant)**				
Left ventricular ejection fraction (%)	21.2 ± 6.0	21.3 ± 5.7	20.9 ± 7.0	0.806
Indexed LV diastolic volume (ml/m^2)	138.9 ± 43.9	133.7 ± 39.1	151.8 ± 53.1	0.168
Right ventricular disfunction	18 (24.0)	12 (22.2)	6 (28.5)	0.781
**Mitral regurgitation**				
Mild	28 (37.0)	22 (40.7)	6 (28.6)	0.510
Moderate	31 (40.0)	22 (40.7)	9 (42.8)	
Severe	16 (23.0)	10 (18.5)	6 (28.6)	
**Tricuspid regurgitation***				
Mild	50 (67)	33 (61.1)	17 (80.9)	0.078
Moderate	20 (28)	18 (33.3)	2 (9.5)	
Severe	5 (5.0)	3 (5.6)	2 (9.5)	
**Aortic regurgitation**				
None	50 (67.0)	39 (72.2)	11 (52.4)	0.086
Mild	24 (31.0)	15 (27.8)	9 (42.8)	
Moderate	1 (2.0)	0	1 (4.8)	
**Right catheterization**				
Cardiac output (l/min)	4.2 ± 0.8	4.1 ± 0.8	4.4 ± 0.8	0.307
Cardiac Index (l/min/m^2)	2.2 ± 0.4	2.2 ± 0.4	2.3 ± 0.4	0.319
Oxygen consumption (ml/min)	273.3 ± 39.9	272.9 ± 29.8	274.4 ± 57.9	0.915
Pulmonary artery saturation (%)	57.2 ± 8.8	56.8 ± 9.7	58.2 ± 6.7	0.615
Systolic pulmonary artery pressure (mmHg)	45.2 ± 12.9	44.3 ± 12.1	47.5 ± 14.8	0.413
Diastolic pulmonary artery pressure (mmHg)	25.7 ± 8.1	25.8 ± 7.8	25.8 ± 9.0	0.995
Mean pulmonary artery pressure (mmHg)	32.9 ± 9.7	33.0 ± 8.6	32.6 ± 12.3	0.907
Pulmonary capillary wedge pressure (mmHg)	20.7 ± 6.4	20.3 ± 6.1	21.5 ± 7.3	0.531
Pulmonary vascular resistance (WU*mq)	6.1 ± 4.4	5.8 ± 2.8	6.6 ± 6.7	0.635
Diastolic pulmonary gradient	4.8 ± 6.5	5.4 ± 5.4	3.6 ± 8.4	0.307

Values are reported as mean ± DS and number (N), and percentage (%). GIB: Gastrointestinal Bleeding; LV: Left Ventricular.
